# Men, Mental Health and Elite Sport: a Narrative Review

**DOI:** 10.1186/s40798-018-0175-7

**Published:** 2018-12-19

**Authors:** Gary Souter, Robin Lewis, Laura Serrant

**Affiliations:** 1 Sheffield Hallam University, Faculty of Health & Wellbeing, F426 Robert Winston Building, 11-15 Broomhall Road, Sheffield, S10 2BP UK; 2 Sheffield Hallam University, Faculty of Health & Wellbeing, M104 Mundella House, 34 Collegiate Crescent, Sheffield, S10 2BP UK; 3 Manchester Metropolitan University, Brooks Building Manchester Campus, Manchester, UK

**Keywords:** Male, Mental health, Elite performance, Depression, Stress, Injury

## Abstract

Mental health in elite sport is receiving more publicity due to an increase in male athletes sharing their personal experiences. Sports injury is recognised as the major risk factor for psychological distress amongst male athletes, although anecdotally this may be that athletes are more likely to discuss their emotional wellbeing when related to the injury they are experiencing. Stress can be amplified within elite sport and the pressure they experience in relation to competition and performance can be exacerbated by adverse life events. This ongoing stress does not end when their sporting career does, it can follow them into retirement. The physical and psychological demands placed upon them by the sporting environment may predispose athletes to developing depression. As an athlete’s symptoms of mental illness intensify, their performance can be negatively affected leaving them vulnerable and exposed to further symptoms of common mental disorders. The pressure of performance can also expose male athletes to overtraining syndrome which can be difficult to distinguish from depression. Male athletes are more vulnerable to eating disorders compared with males in the general population and they do have anxieties, particularly around their bodies, but find it difficult to disclose their concerns. In addition to this, male athletes are more likely to use substances, including opioids to improve both sport and non-sport performance.

Despite the prevalence of common mental disorders in male athletes, stigma still exists, and although some athletes discuss their issues publicly after their career has ended, the majority of athletes prefer to remain silent. There remains a view that athletes who seek help for psychological problems may be seen as weak. Although there is an improvement in help-seeking attitudes within elite sport, further research and education is needed to encourage men to talk about their mental health, share their experiences and to enjoy a greater sense of emotional wellbeing.

## Key points

- Major negative life events, including injury, may increase the risk of mental ill-health in elite athletes.
- Male athletes need to be encouraged to discuss their emotions, concerns and anxieties.
- There should be further education and awareness of mental health issues aimed at elite male athletes.

## Background

Professional sportsmen are recognised for the stress and strain that they put their bodies through and it is widely accepted that they will encounter physical injury at some point. There is a societal view that sport is about winning and competing, and the focus is predominantly about individual or team performance. Male athletes can be viewed as superhuman and the impact of their emotional wellbeing is frequently overlooked. Mental health in elite sport is receiving more publicity due to an increase in male athletes sharing their personal experiences. The fields of sports psychology and sports psychiatry are rapidly developing areas aimed at understanding, diagnosing, treating and rehabilitating athletes. Sport psychology focuses upon the use of mental skills training within the sporting domain [1] and sports psychiatry aims to optimise the health of athletes, improve athletic performance, as well as managing psychiatric symptoms or disorders of athletes [2].

The aim of this review is to explore the issues affecting men, and their mental health in the context of elite sport.

The term mental health encapsulates a wide variety of meanings. Mental health can be seen in both a positive and negative light. Mental ill health covers a range of disorders and diagnoses many of which can have a debilitating impact on the sufferer. In contrast to this, mental wellbeing can be defined as when an individual realises his or her own potential, can cope with the normal stresses of life, can work productively and fruitfully and is able to make a contribution to her or his community [3]. One in four people in the UK will be affected by mental illness in any year with the most common being depression and anxiety [3]. Common mental disorders include depression, generalised anxiety disorder, panic disorder, phobias, social anxiety disorder, obsessive-compulsive disorder and post-traumatic stress disorder [4]. For the purpose of this review, the focus will be based on the following areas; injury, stress, depression, anxiety, overtraining, disordered eating, substance abuse, help seeking and mental toughness.

## Main Text

### Injury

One of the most recognised risk factors for psychological distress amongst male athletes has been sports injury [5]. Major negative life events, including injury, may increase the risk of mental ill-health in elite athletes [6]. Injuries are particularly found to be associated with depression in athletes [7]. Symptoms of common mental disorders amongst both current and former athletes, including depression and anxiety, were found to be related to several determinants, particularly injury [8], and former athletes who had suffered from several severe injuries or surgeries were 2–7 times more likely to report symptoms of common mental disorders than former athletes without injury or surgery. Injured athletes have higher depression scores than non-injured athletes [7]. This may be due to the view that injured athletes are more likely to discuss their emotions related to the injury they are experiencing. It is suggested that professional footballers who had sustained one or more severe joint injuries during their career were three to four times more likely to report distress compared to footballers who had not suffered from severe joint injuries during their career [9]. Athletes suffer from a higher risk of injuries and 80% of the time, athletes attending for treatment on their injury also discussed their psychological issues caused by the injury [5]. In a focus group study, negative emotions such as depression, sadness and anger were potential outcomes for both short- and long-term injuries [10].

It is also worth considering the risk of head injuries and the link between head injuries and depression [10]. The concern of head injuries and particularly concussion is a topic of continuing debate in the United States (US). The National Football League (NFL) players regularly experience repeated trauma to their heads during each match they play. The limitations of this research need to be acknowledged as the evidence includes self-reports and self-selecting samples. There is a significant relationship between the number of self-reported concussions and self-reported depressive symptoms in retired NFL players [11]. Concussion and multiple brain injuries can be related to depression [12, 13]. Former NFL players took legal action against the NFL due to concerns that repeated head injuries and concussion were causing chronic traumatic encephalopathy (CTE) which can only be diagnosed after death. A study found that 99% of professional NFL athletes had a disease associated with head injuries [14]. Out of a sample of 202 players, 87% were found to have traces of CTE. Although these results need to be viewed with caution as the brains of the sample were donated by concerned family members, however the awareness of the impact of head injuries and the link to depression, Alzheimer’s disease and CTE is certainly on the increase. Whatever the type or cause of sport injury and the physical impact it will have on the athlete, it will evidently serve as a psychological stressor for the athlete [15] **.**

### Stress

A professional sports career can include in excess of 640 stressors that may induce common mental disorders [16]. Even if mental ill health was evident prior to involvement in sport, it may become amplified when athletes are faced with the stressors that are associated with elite sports [17]. Within elite sport, athletes face an extreme load of physical training and psychological stress [15]. There is a need to consider competition-induced stress either prior, during or immediately after competition [18], and include factors of an athlete’s sporting life including training, rehabilitation, team meetings and contract negotiations. Professional sportsmen are not immune from stress. They are vulnerable to profession-specific stressors as well as life events similar to the general population. The stress players experience does not end when their playing career does, it follows them into retirement. For example, retired professional footballers are as likely as anyone to be exposed to stressors especially major life events [9].

Retirement is a major life event that can have a significant impact when an athlete’s career is over. This can result in changes to an athlete’s interpersonal relationships, roles and daily routines. Athletes experience conflict in relation to deciding to continue in or retire from their sport and whether this would alleviate their depressive symptoms. They can develop feelings of worthlessness, and a mentality of cannot live with it, cannot live without it [19]. Career termination from elite sport has been associated with maladaptive coping strategies, depression, anxiety, increased hostility and anger and substance abuse [5]. Athletes who transition from active sport to retirement can experience a loss of identity, social networks and public identity [20]. Frequently, athletes do not recognise the impact of retirement until they are going through the process and involuntary career termination, such as injury, is more likely to have a negative impact upon an athlete’s mental health compared to voluntary career termination, which is a personal decision to retire [5]. There appears to be an unwillingness amongst younger athletes and those who perceive themselves to have a significant amount of time before they retire to develop concrete plans about their future career prior to their retirement [21]. Athletes with high levels of athletic identity will derive greater satisfaction from the athletic aspects of their lives [22], although athletes going into retirement with high levels of athletic identity are likely to experience adjustment problems [23]. Athletes who maintain a strong athletic identity up to the point of retirement may be vulnerable to career transition difficulties [24], and athletes whose identity is based on participation and success in sport are more vulnerable to psychological issues such as depression [25].

An autobiographic study of top level athletes found that several of the athletes reported external stressors including bereavement, family health concerns, relationship breakdown and multiple injuries [19]. Internal stressors such as self-loathing, social anxiety and self-criticism were also reported. Life stressors were also common in a study looking at suicide amongst test cricketers [26]. Health concerns, relationship breakdown and financial difficulties were cited as being external factors. The same study highlights that there have not been any suicides amongst test cricketers since 1998. This could be attributed to the fact that professional cricket has developed a culture where players are encouraged to seek support for the stress they endure, although mental health issues are still prevalent within cricket. The Professional Cricketer’s Association (PCA) have a benevolent fund and wealth of resources to support their members with any issues that they may experience [27]. There is a high prevalence of up to 38% for common mental disorders amongst current professional cricketers [28]. Cricketers’ career dissatisfaction led to an increase in distress and adverse life events led to reported symptoms of distress, anxiety and depression.

A study analysed sources of stress for Professional Australian Footballers [18]. Following interviews and focus groups, they identified 77 sources of stress and grouped them into 6 key themes. Two of these themes appear key in comparison to other studies. One theme regarding worries about performance expectations and standards is supported by the view that athletes experience a unique range of stressors including managing ongoing competitive pressures to perform [6], and the pressure to deliver peak performance [29]. Depressive symptoms can be related to the failure to achieve performance-related goals [30]. Another theme focused on the negative aspects of interpersonal relationships. This could relate to conflict with trainers, partners and family [15], or mistakes by teammates or a feeling of letting teammates down [31].

### Depression

Athletes may be more predisposed to depression compared to the general population and suggests that this is due to the physical and psychological demands placed upon them by the sporting environment [32]. They are exposed to similar stressors as the general population with athletes experiencing bereavement, health concerns and relationship breakdowns [33]. Elite athletes are vulnerable to developing depressive symptoms due to their high status and extreme pressure that they experience [34]. Publicly evaluated performance and a perceived acceptance in an elite environment which is dependent upon results can be a vulnerability factor for depression [35]. This sport-related stress when combined with other factors including moving away from home, risk taking behaviours and disordered eating may increase the risk of athletes developing common mental disorders [10].

There are a number of factors that increase an athlete’s risk of depression compared with the general population [5]. These factors include injury, termination and performance pressures. There is a significant relationship between an athlete’s depressive symptoms and performance [36], and as an athlete’s depressive experiences intensified, their performances were negatively affected by their symptoms [19], although sometimes other symptoms of mental disorders such as anxiety can occur before depression.

There are mixed results within the literature examining athletes and depression. In US college athletes, 21.4% of athletes self-reported clinical symptoms of depression [37], and in Australian athletes, 46.4% of athletes experienced symptoms of at least one common mental disorder including 27.2% for depression [7]. The literature confirms that depression does exist within male athletes and there are a variety of factors both related to being an athlete and also factors comparable to the general population.

### Anxiety

Anxiety in athletes can be both facilitative and debilitative. Elite athletes can view symptoms of anxiety as performance enhancing [38]. In some sports competition, anxiety is considered as normal, although athletes who experience excessive levels of competition anxiety can experience negative consequences such as impaired performance [39]. In some sports, for example cycling, pre-race anxiety is common, and anxiety can be both helpful and unhelpful to performance [40]. Competition anxiety can be linked to self-confidence and athlete’s self-confidence levels can fluctuate closer to the event and depending upon the level of competition. These competitive anxiety symptoms can become habitual and ingrained within the athlete [41]. High levels of self-confidence can help to protect athletes from interpreting anxiety symptoms as debilitative and change their emotional response to a positive state [42]. Younger athletes can be more susceptible to competition anxiety and social phobia in comparison to older athletes [43]. This is supported by a study examining the prevalence of anxiety and depression in Swiss football players. They found that male under-21 footballers had anxiety scores comparable to the general population, although footballers over 21 had significantly lower average anxiety scores [44]. In comparison, a study examining the prevalence of anxiety and depression of elite rugby league players found that the prevalence of generalised anxiety disorder symptoms ranged from 10.1 to 14.6% which is in excess of the general population rate of 5% [45].

There is evidence to suggest that athletes who have experienced concussion may be at an increased risk of anxiety symptoms. Also, elite athletes may be susceptible to concussion due to pre-existing mental health symptoms, for example preoccupation, increased distraction or emotional dysregulation [46].

### Overtraining

Another factor that can significantly impact upon an athlete’s mental health is overtraining, or the overtraining syndrome (OTS). This is where the human body attempts to cope with physiological and other stressors including physical training, sleep loss, exposure to environmental stresses (e.g., exposure to heat, high humidity, cold, high altitude), occupational pressures, change of residence and interpersonal difficulties [47]. OTS can be difficult to distinguish from depression as some of the symptoms such as fatigue, insomnia, appetite change, weight loss, lack of motivation and concentration difficulties overlap with each other [48]. OTS can occur when an athlete undergoes a rigorous training schedule and has an insufficient recovery period caused through other sources of non-training stress. Overtraining is a staleness in which athletes do not recover from previous exhaustion despite a recovery period of at least 2 weeks [15]. This may lead to maladaptive responses and in turn have a significant impact upon performance [49]. The prevalence of overtraining in elite athletes has been reported at between 20 and 60% and distance runners are the most severely affected [50]. An athlete’s addictive behaviour may be responsible for OTS due to their sustained and excessive training [51], also overtraining could be interpreted as a form of self-harm in that it may be that overtraining served the function of a cry for help and a maladaptive attempt to communicate internal distress [35].

The extreme of OTS is burnout, which has been reported in approximately 10% of elite athletes [50]. When an athlete is over trained, their motivation remains, although when athletes suffer from burnout, they typically experience chronic fatigue, poor sleep patterns, episodes of depression and helplessness [52]. It is therefore not surprising that their performance is negatively affected.

### Disordered Eating

Disordered eating occurs on a continuum ranging from dieting and restrictive eating at one end, to abnormal eating behaviour, and clinical eating disorders at the other end of the continuum [53]. Male elite athletes are more vulnerable to eating disorders than males in the general population [54, 55]. A study on dieting and disordered eating found that 59% of the young male elite athletes were dissatisfied with their body, 19% were dieting and 11% had disordered eating [56]. The prevalence of disordered eating was 10%, 17% and 42% for endurance, weight class and antigravitations sports, respectively [56].

Elite athletes often embody the concept of physical perfection and they can often experience pressure to achieve an ‘ideal’ body type [57], and they can experience the stress of constantly denying hunger, obsessing about food, agonising over body weight and fearing high body weight which can be mentally exhausting.

Symptoms of eating disorders can occur more frequently in sports where low body weight enhances performance, which are subdivided in weight classes or in which weight is advantageous [58]. Elite athletes involved in weight-sensitive sports are at greater risk of developing eating disorders than the general population [59], and male athletes who compete in weight class sports such as wrestling and rowing are at an increased risk whilst male athletes who compete in swimming and figure skating are at a reduced risk for eating disorders [60]. Body weight is also important in sports where aerodynamics and reduced resistance or drag is important such as cycling and swimming [57]. Male athletes do have body anxieties but find it difficult to disclose their health-related concerns [61].

### Substance Abuse

Substance abuse and substance addiction may be a concern within elite sport. Athletes often use substances to assist performance. The reasons they may abuse substances include stress relief, psychological dependence, negative emotions reduction and tolerance/withdrawal [62].

Higher rates of alcohol consumption and binge drinking may occur in athletes [63], and athletes may indulge in risk taking behaviours such as hazardous drinking [64]. Substance abuse amongst elite adolescent athletes and alcohol consumption is a cause for concern and binge drinking was performed by every third male athlete [65]. Current athletes consume more alcohol than non-athletes and alcohol consumption was more common amongst team sports athletes than other sports [66]. Cannabis appears to be the second most widely used drug after alcohol, although many elite athletes deny its use [67]. Men are more likely to use cannabis to improve both sport and non-sport performance [68], and male adolescents participating in organised sports are more likely to abuse opioids compared with both non-athlete peers and females [69]. Elite athletes may also become addicted to stimulants for performance enhancement, although they may be a range of adverse effects including anxiety, insomnia, irritability and weight loss [70]. Risk taking behaviour such as gambling within elite athletes is currently under-researched; however, gambling is widespread amongst professional athletes with at least 56% having gambled at least once in the previous year, which is much greater than the general population across Europe [71]. Anecdotal evidence suggests that gambling addiction is particularly problematic within professional football; however, further research needs to be commissioned to understand the issues in greater detail.

### Help Seeking

It is clear from the literature that elite male athletes do experience a range of common mental disorders and stressors that are either created externally or internally. However, it appears that athletes prefer to discuss their issues or talk publicly after their elite career has ended. This is supported by the large number of sporting autobiographies released each year. A factor behind this reluctance to discuss issues during their playing career could be related to the stigma around mental health. Stigma has been highlighted as a barrier to help seeking in athletes [10]. The prevalence of common mental disorders in elite sport is underreported because of stigma [72]. There is a view that athletes who do seek help for psychological problems may be seen as weak by other athletes and coaches [73]; however, it is important to highlight that it is okay for men to talk about problems and feelings—it should not be seen as a sign of weakness, rather it is a strength [74].

A focus group study found that athletes would be worried about others finding out if they were seeking help for a mental health problem [10]. Stigma could be seen as a strategy to protect one’s survival in the team, as well as to cast out individuals who threatened the team’s success [75]. Athletes are used to being in the spotlight and may have ‘situational narcissism’ which can make it difficult for them to seek or accept assistance [76]. They also suggest that athletes may not easily discuss their emotions as they have been taught to work through pain.

There is starting to be a shift in help-seeking attitudes within elite sport. As previously mentioned, Cricket have put an emphasis upon player welfare as have Rugby League in particular. Rugby League in conjunction with National Health Service (NHS) professionals created State of Mind Sport (SOMS) followed the death of Terry Newton, an international rugby league player. SOMS has been supported by the Time to Change campaign, Mind, the Samaritans and the suicide prevention charity PAPYRUS, which has enabled former players to access their training programmes [74]. The supportive work that cricket and rugby are doing to break down barriers and challenge stigma are an important influence upon this review.

### Mental Toughness

To understand mental health in elite sport, it is worth considering the concept of mental toughness and how that may impact upon an athlete. There is a belief that mental toughness and mental health are contradictory in elite sport [17]. The stigma regarding seeking help may jeopardise an athlete’s place in the team or their contract to compete. Therefore, seeking help to get better may have a detrimental effect in an athlete’s sporting role. Athletes were asked to define mental toughness in their own words and found that mental toughness provided the performer a psychological advantage over their opponents [77]. They also found that mentally tough performers consistently remained determined, focused, confident and in control of the pressures and demands of their sport. Mental toughness may represent a positive indicator of mental health, or facilitate its attainment, rather than be at odds with it [78]. Athletes and coaches may show an interest in programs that are termed mental toughness development in order to engage in discussions around mental health. State of Mind Sport have developed sessions for rugby players and used the language of ‘mental fitness’ to draw them in [74].

In terms of football, coaches were united in the notion that mental toughness is a key ingredient to a successful sporting career [79]. This was expressed in the notion that ‘It’s the difference between making or breaking careers’, and a significant influencer of team selection. Communication is important in mental toughness; mentally tough players would communicate positively by encouraging and activating their teammates, whereas negative communication either towards oneself, teammates or opponents were indicated as a sign of mental weakness [80]. Mentally tough academy players had a desire to achieve, a love for the sport alongside a commitment to excellence that translated passion into success [81].

## Conclusion

Major negative life events, including injury, may increase the risk of mental ill-health in elite athletes [6]. They are vulnerable to profession-specific stressors as well as life events similar to the general population. Although male athletes will discuss psychological issues whilst they are undergoing treatment for injury, more work needs to be done to encourage them to open up and discuss their emotions, concerns and anxieties. Male athletes are under constant performance pressure in relation to the sport, although the literature suggests that they do not prioritise their emotional wellbeing. Many of them also have the additional pressure of living their life in the public eye which can be an additional challenge that some athletes are not used to. It can become a vicious circle when men suffer emotionally, it can impact on their personal and sporting life; this can then lead to performance issues and in turn increase their injury risk. And as the literature suggests, injuries can then increase the risk of mental health issues and adverse behaviours. Although there are a number of help-seeking campaigns particularly supported on social media, there should be further education and awareness of mental health issues aimed at elite male athletes. Male athletes cannot be mentally tough all of the time but if they are supported and encouraged to seek help and share their experiences, this will significantly improve their mental fitness and give them a greater sense of emotional wellbeing.

## Abbreviations

CTE: Chronic traumatic encephalopathy
NFL: National Football League
NHS: National Health Service
OTS: Overtraining syndrome
PCA: Professional Cricketer’s Association
SOMS: State of mind sport
UK: United Kingdom
US: United States
